# Downregulation of protein kinase CK2 activity induces age-related biomarkers in *C. elegans*

**DOI:** 10.18632/oncotarget.16939

**Published:** 2017-04-07

**Authors:** Jeong-Hwan Park, Joo-Hyun Lee, Jeong-Woo Park, Dong-Yun Kim, Jeong-Hoon Hahm, Hong Gil Nam, Young-Seuk Bae

**Affiliations:** ^1^ School of Life Sciences, BK21 Plus KNU Creative BioResearch Group, College of Natural Sciences, Kyungpook National University, Daegu, Republic of Korea; ^2^ School of Life Sciences, College of Natural Sciences, Kyungpook National University, Daegu, Republic of Korea; ^3^ Center for Plant Aging Research, Institute for Basic Science, Daegu, Republic of Korea; ^4^ Department of New Biology, DGIST, Daegu, Republic of Korea

**Keywords:** C. elegans, protein kinase CK2, longevity, daf-16, Gerotarget

## Abstract

Studies show that a decrease in protein kinase CK2 (CK2) activity is associated with cellular senescence. However, the role of CK2 in organism aging is still poorly understood. Here, we investigated whether protein kinase CK2 (CK2) modulated longevity in *Caenorhabditis elegans*. CK2 activity decreased with advancing age in the worms. Knockdown of *kin-10* (the ortholog of CK2β) led to a short lifespan phenotype and induced age-related biomarkers, including retardation of locomotion, decreased pharyngeal pumping rate, increased lipofuscin accumulation, and reduced resistance to heat and oxidative stress. The long lifespan of *age-1* and *akt-1* mutants was significantly suppressed by *kin-10* RNAi, suggesting that CK2 acts downstream of AGE-1 and AKT-1. *Kin-10* knockdown did not further shorten the short lifespan of *daf-16* mutant worms but either decreased or increased the transcriptional activity of DAF-16 depending on the promoters of the target genes, indicating that CK2 is an upstream regulator of DAF-16 in *C. elegans*. *Kin-10* knockdown increased production of reactive oxygen species (ROS) in the worms. Finally, the ROS scavenger N-acetyl-L-cysteine significantly counteracts the lifespan shortening and lipofuscin accumulation induced by *kin-10* knockdown. Therefore, the present results suggest that age-dependent CK2 downregulation reduces longevity by associating with both ROS generation and the AGE-1-AKT-1-DAF-16 pathway in *C. elegans*.

## INTRODUCTION

The nematode *Caenorhabditis elegans* has been widely used as a model for exploring the mechanisms underlying aging. A crucial pathway influencing *C. elegans* aging is the insulin/insulin-like growth factor (IGF)-1 signaling (IIS) pathway. Key components of the IIS pathway are *daf-2*/IGF receptor (IGFR), *age-1*/phosphoinositide 3-kinase (PI3K), and *akt-1*/AKT-1/2 [1–4]. The transcription factor DAF-16/FoxO lies downstream of the IIS pathway [5, 6]. DAF-2/IGFR activation leads to the activation of AGE-1/PI3K, which in turn activates AKT, resulting in phosphorylation and cytoplasmic sequestration of DAF-16/FoxO [7]. Inhibition of DAF-2/IGFR or AGE-1/PI3K allows translocation of DAF-16/FoxO into the nucleus, where DAF-16/FoxO activates the transcription of pro-longevity genes, including antioxidant and thermotolerant genes [8, 9].

Cellular senescence, which is defined as an irreversible arrest at the G1 phase of the cell cycle, is an important tumor suppression process *in vivo*. Because the number of senescent cells increases with organism aging, it is widely believed that cellular senescence plays an important role in aging. We have previously shown that protein kinase CK2 (CK2), which is a ubiquitous serine/threonine kinase, is downregulated during replicative senescence in human lung fibroblast IMR-90 cells and in aged rat tissues [10]. CK2 inhibition induces premature senescence in IMR-90 cells, human colon cancer HCT116 cells, and breast cancer MCF-7 cells. The p53-p21^Cip1/WAF1^ pathway is required for senescence induced by CK2 inhibition [11]. Reactive oxygen species (ROS) production and p53 acetylation by downregulation of NAD^+^-dependent protein deacetylase SIRT1 serve as upstream activators of p53 stabilization in senescent cells due to CK2 inhibition [12, 13]. Inhibition of PI3K, AKT, and mammalian target of rapamycin (mTOR), as well as ectopic expression of FoxO3a, attenuated ROS production and senescence in CK2-downregulated cells, indicating that the PI3K-AKT-mTOR pathway as well as FoxO3a are involved in CK2 inhibition-mediated ROS generation [14, 15].

The CK2 holoenzyme is a heterotetramer composed of two catalytic (α and/or α′) subunits and two regulatory β subunits. The β subunit stimulates the catalytic activity of the α or α′ subunit, thereby mediating tetramer formation and substrate recognition [16]. The expression level of CK2 is greatly enhanced in a variety of tumor or leukemic cells [17–19]. The biological role of CK2 has been extensively studied in diverse organisms. KIN-3 and KIN-10, the *C. elegans* orthologs of CK2α and CK2β, respectively, are known to be expressed ubiquitously in multiple tissues [20, 21]. In this study, we characterized the role of CK2 as a positive regulator of longevity in worms. We found that CK2 activity decreases with advancing age and that downregulation of CK2 activity by *kin-10* RNAi causes expression of age-related biomarkers and lifespan reduction. This study provides the first evidence that age-dependent downregulation of CK2 activity may be a possible regulator of lifespan in *C. elegans*.

## RESULTS

### In *C. elegans*, CK2 activity is downregulated with advancing age, and *kin-10* knockdown reduces lifespan and pumping rate

**Figure 1:**
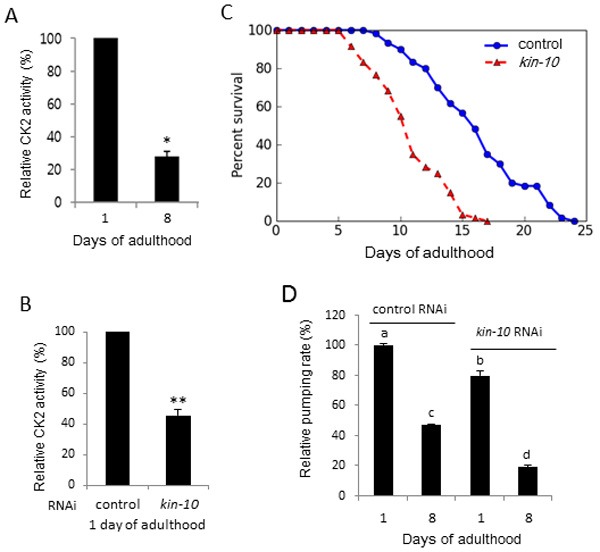
In *C. elegans*, CK2 activity was downregulated with advancing age, and *kin-10* knockdown reduced lifespan and pumping rate Age-synchronized L4 larvae were fed on empty vector control (L4440) or *kin-10* RNAi plates under standard conditions. **A**. Age-dependent decrease in CK2 activity. Lysates from worms at days 1 and 8 of adulthood were utilized in kinase assays using a specific CK2 substrate peptide. ^32^P incorporation into the substrate peptide was measured by scintillation counting. **B**. *Kin-10* knockdown reduced CK2 activity in worms. Lysates from worms at day 1 of adulthood fed with *kin-10* RNAi or control RNAi were utilized in kinase assays using a specific CK2 substrate. ^32^P incorporation into the substrate peptide was measured by scintillation counting. **C**. *Kin-10* RNAi reduced the lifespan of worms. Viability was scored as movement away from pick touch at the indicated days. Representative data from three independent RNAi experiments are shown (*n* = 60 per condition). **D**. *Kin-10* knockdown reduced pharyngeal pumping rate. Each data point represents the mean of relative pumping rate per minute (*n* = 20 per condition). Values indicate mean ± SEM. *P < 0.05; **P < 0.01. Bars that do not share a common letter (a, b, c, d) are significantly different among the groups at *P* < 0.05. All experiments in this figure were carried out at 21 °C.

We first examined whether CK2 catalytic activity *in C. elegans* was modulated with advancing age. The phosphotransferase activity of CK2 decreased by 70% in worms at day 8 compared to worms at day 1 (Figure 1A). To investigate whether CK2 regulates the longevity of *C. elegans*, we compared the lifespan of worms treated with *kin-10* RNAi with that of worms treated with empty vector control (L4440) RNAi. The phenotype of *kin-3* RNAi was lethal under our experimental conditions. The growth rate of *kin-10* RNAi worms was almost the same as that of wild-type worms (data not shown). CK2 phosphotransferase activity in *kin-10* RNAi worms was reduced by approximately 50% compared to that of control RNAi worms (Figure 1B). After *kin-10* RNAi treatment, the maximum lifespan of the worms was shortened from 24 to 17 days, and the median lifespan decreased from 16 to 10 days (Figure 1C; see also Supplemental Figure 1). We also carried out lifespan assays using worms with *kin-10* RNAi only during adulthood and observed similarly shortened lifespans (Supplemental Figure 2A). This result eliminates the possibility that downregulation of *kin-10* during larval development harmfully affected the health of worms and consequently decreased their longevity during adulthood. We next investigated the effect of *kin-10* knockdown on the worms’ pharyngeal pumping. Compared with that of worms at day 1 of adulthood, the pumping rate was much lower in worms at day 8 of adulthood. However, pumping rates further decreased in worms fed with *kin-10* RNAi (Figure 1D). Therefore, the results suggest that CK2 may function as a positive regulator of aging in *C. elegans*.

### *Kin-10* knockdown increases lipofuscin and fat accumulation in *C. elegans*

**Figure 2:**
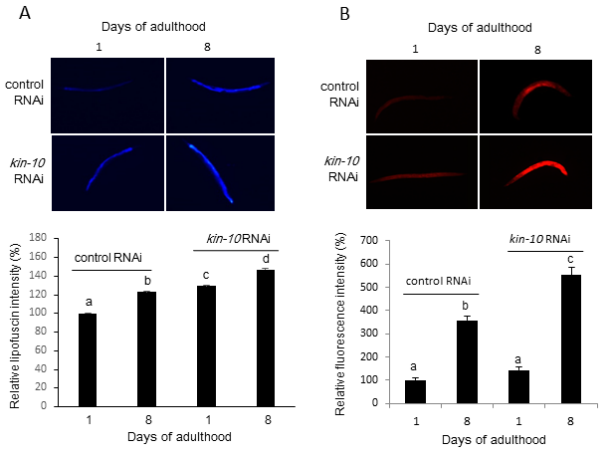
*Kin-10* knockdown increased lipofuscin accumulation and Nile red staining in *C. elegans* **A**. Knockdown of *kin-10* increased lipofuscin accumulation. Representative autofluorescence images of worms at day 1 or 8 of adulthood (upper panel). The fluorescence intensity (bottom panel) was quantified using the ImageJ software by determining the average pixel intensity (*n* > 80 per condition). **B**. Representative Nile red staining images (upper panel) of worms at day 1 or 8 of adulthood and relative fluorescence intensity (bottom panel) are shown. The fluorescence intensity was quantified using ImageJ software by determining the average pixel intensity (*n* = 20 per condition). Values indicate mean ± SEM. Bars that do not share a common letter (a, b, c) are significantly different among the groups at *P* < 0.05. All experiments in this figure were carried out at 21 °C.

Increased lipofuscin and fat accumulation are also associated with accelerated aging [1]. To investigate the effect of *kin-10* on lipofuscin accumulation in worms, lipofuscin accumulation was determined by autofluorescence [22]. Autofluorescence significantly increased with age but also increased in *kin-10* RNAi worms relative to control RNAi worms (Figure 2A). To exclude the effect of *kin-10* on lipofuscin accumulation during development, we examined worms fed with *kin-10* RNAi only during adulthood and observed similar lipofuscin accumulation (Supplemental Figure 2B). Nile red staining indicated that *kin-10* RNAi stimulated fat accumulation in the worms (Figure 2B). Therefore, these results suggest that *kin-10* downregulation induced biomarkers of aging in *C. elegans*.

### *Kin-10* knockdown decreases stress resistance and motility in *C. elegans*

**Figure 3:**
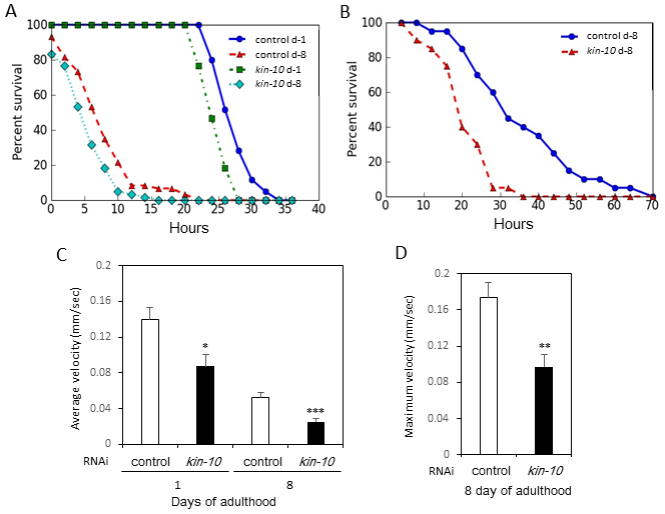
*Kin-10* knockdown decreased resistance against heat and oxidative stress and motility in *C. elegans* **A**. *Kin-10* knockdown decreased resistance against heat stress at 35 °C. Worms at day 1 (d-1) or 8 (d-8) of adulthood were scored every 2 h for viability (*n* = 60 per condition). **B**. *Kin-10* knockdown decreased resistance against oxidative stress. Survival curves of worms fed with control or *kin-10* RNAi after exposure to 3 mM H_2_O_2_ for 5 h. Worms at day 8 (d-8) of adulthood were scored every 4 h for viability (*n* = 40 per condition). C and D. *Kin-10* RNAi decreased average velocity **C**. and maximum velocity **D**. of worms. The average velocity and maximum velocity of individual worms were measured at day 1 or 8 of adulthood (*n* > 13 per condition). Data are shown as the means ± SEM. *P < 0.05; **P < 0.01; ***P < 0.001.

Aged worms display a general decrease in resistance to diverse stresses, including heat stress and oxidative stress [1]. We performed a thermotolerance assay and found that not only aging but also *kin-10* RNAi decreased resistance to heat stress at 35 °C compared to control treatment with the empty vector (Figure 3A). Resistance to oxidative stress (3 mM H_2_O_2_) was also decreased by *kin-10* RNAi (Figure 3B). Because all animals display age-related decline in physical ability, we measured the worms’ body movement. As shown in Figure 3C, *kin-10* RNAi decreased the average worm velocity by approximately 35% and 50% compared to control RNAi at day 1 and 8 of adulthood, respectively. In addition, at day 8 of adulthood, *kin-10* knockdown decreased the worms’ maximum velocity by 47% compared to control RNAi (Figure 3D). Taken together, these results also suggest that *kin-10* downregulation induced biomarkers of aging in *C. elegans*.

### CK2 regulates lifespan downstream of AGE-1 and AKT-1 in *C. elegans*

**Figure 4:**
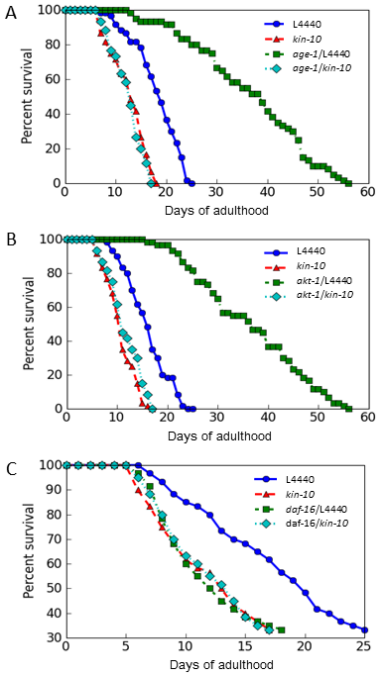
Effect of *kin-10* knockdown on the lifespan of *age-1*, *akt-1*, and *daf-16* single mutant worms *Age-1(hx546)* **A**., *akt-1(mg144)* **B**., and *daf-16(mu86)* **C**. single mutant worms were fed on the control (L4440) or *kin-10* RNAi plates under standard conditions. *n* = 60 per condition (A and B); *n* = 40 per condition (C). Viability was scored as movement away from pick touch at the indicated days. *Kin-10* knockdown successfully suppressed the longevity extension by *age-1(hx546)* and *akt-1(mg144)* mutations, but did not synergistically shorten the lifespan of *daf-16(mu86)* mutant. All experiments in this figure were carried out at 21 °C.

The IIS pathway is a critical modulator of longevity, along with DAF-16/FoxO [1, 9]. To define the role of CK2 in the aging pathway, we examined the genetic interactions between CK2 and the IIS pathway. Our previous studies demonstrated that the AGE-1/PI3K-AKT pathway is involved in CK2 inhibition-mediated cellular senescence in human cells [14]. Therefore, we used the *age-1(hx546)* and *akt-1(mg144)* single mutants. There was a significant increase in the lifespans of both *age-1(hx546)* and *akt-1(mg144)* single mutants. The maximum and median lifespans of these mutant worms were extended to 56 and 37 days, respectively. However, *kin-10* RNAi completely suppressed the longevity extension effect of the *age-1(hx546)* and *akt-1(mg144)* mutations. Upon *kin-10* RNAi treatment, the maximum and median lifespans of the mutant worms were shortened to levels similar to those of wild-type worms fed with *kin-10* RNAi (Figure 4A and 4B). Thus, these results suggest that CK2 is associated with the IIS pathway as a downstream regulator of AGE-1 and AKT-1 in *C. elegans*.

### *Kin-10* knockdown in *C. elegans* either decreases or increases DAF-16 activity depending on the promoter of the target genes

**Figure 5:**
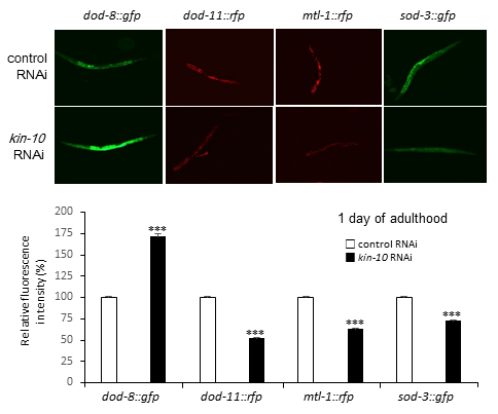
*Kin-10* knockdown either decreases or increases the transcriptional activity of DAF-16 depending on the target genes in *C. elegans* Worms with integrated *sod-3::gfp*, *mtl-1::rfp*, *dod-11::rfp*, and *dod-8::gfp* reporter constructs were fed with *kin-10* RNAi or control (L4440) RNAi at L4 stage for 1 day. One day after RNAi treatment, GFP or RFP fluorescence was imaged with a 10× objective (upper panel). GFP or RFP fluorescence intensity (bottom panel) was quantified using the ImageJ software by determining the average pixel intensity (*n* > 70 per condition). Data are shown as the means ± SEM. ***P < 0.001. All experiments in this figure were carried out at 21 °C.

To examine the role of DAF-16 in the cellular responses subsequent to *kin-10* RNAi, *daf-16* mutant worms were used. The lifespan of *daf-16(mu86)* mutant worms was not synergistically shortened by RNAi inhibition of *kin-10* expression, suggesting the possibility that CK2 and DAF-16 are present in the same signaling pathway regulating longevity (Figure 4C). To examine whether CK2 activity regulates DAF-16 transcriptional activity in *C. elegans*, we measured the fluorescence of several reporter worms that are specific for certain stress pathways, including *sod-3*, which encodes manganese superoxide dismutase (MnSOD), *dod-11*, which encodes sorbitol dehydrogenase, *mtl-1*, which encodes metallothionein, and *dod-8*, which encodes 17 β-hydroxysteroid dehydrogenase [23]. *kin-10* RNAi decreased the fluorescence of *sod-3::gfp*, *dod-11::rfp*, and *mtl-1:rfp* reporter worms compared to the control RNAi. By contrast, *kin-10* RNAi enhanced the fluorescence of the *dod-8*::*gfp* reporter relative to the empty vector RNAi (Figure 5, upper panel). Further densitometric analysis of fluorescence images confirmed that *kin-10* knockdown downregulates the expression of *sod-3*, *dod-11*, and *mtl-1* but upregulates the expression of *dod-8* (Figure 5, bottom panel). These results therefore suggest that in *C. elegans*, CK2 regulates the transcriptional activity of DAF-16 either negatively or positively, depending on the target genes upstream of DAF-16.

### *Kin-10* knockdown stimulates ROS production in *C. elegans*

**Figure 6:**
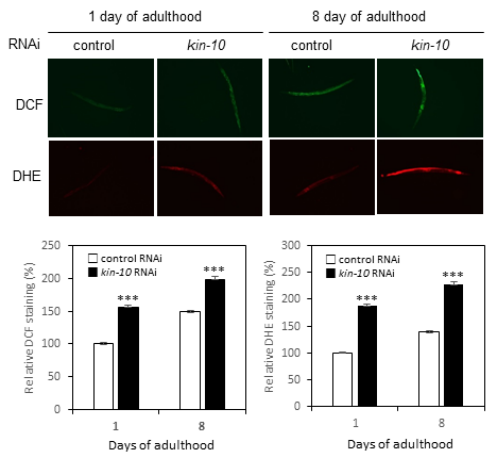
*Kin-10* knockdown stimulates ROS production in *C. elegans* Worms fed with *kin-10* RNAi or control RNAi were incubated with DCFDA or DHE. Representative images at day 1 or 8 of adulthood were obtained at 10× magnification (upper panels). The fluorescence intensity (bottom panels) was quantified using the ImageJ software by determining the average pixel intensity (*n* > 30 per condition). Data are shown as the means ± SEM. ***P < 0.001. All experiments in this figure were carried out at 21 °C.

Excess intracellular ROS cause DNA damage, which can induce cellular senescence. We have also previously shown that ROS play a key role in CK2 inhibition-mediated senescence in human cells [12]. To investigate the role of ROS in aging of *kin-10* RNAi worms, the amount of ROS was quantified using DCFDA and DHE. *Kin-10* RNAi significantly increased the production of ROS in the worms (Figure 6, upper panels). Quantification of ROS levels showed that they increased approximately 1.5- to 2.3-fold depending on age and fluorescent probe in *kin-10* RNAi worms compared with control RNAi worms (Figure 6, bottom panels). Consistently, wild-type N2 worms at day 8 of adulthood exhibited a markedly higher level of ROS than worms at day 1 of adulthood (Figure 6).

### The ROS scavenger NAC counteracts the lifespan shortening and lipofuscin accumulation mediated by *kin-10* knockdown

**Figure 7:**
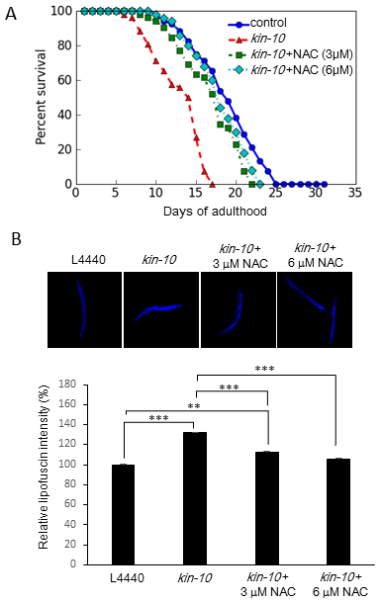
The effect of NAC on lifespan shortening and lipofuscin accumulation in *kin-10* RNAi worms Age-synchronized L4 larvae were fed on empty vector control (L4440) plates or *kin-10* RNAi plates containing NAC (0, 3, or 6 µM) under standard conditions. **A**. Viability was scored as movement away from pick touch at the indicated days. Representative data from three independent RNAi experiments are shown (*n* = 50 per condition). **B**. Representative autofluorescence images of worms at day 1 of adulthood (upper panel). The fluorescence intensity (bottom panel) was quantified using the ImageJ software by determining the average pixel intensity (*n* > 40 per condition). Data are shown as the means ± SEM. **P < 0.01; ***P < 0.001. All experiments in this figure were carried out at 21 °C.

To determine the role of ROS in aging subsequent to *kin-10* knockdown, worms were incubated with N-acetyl-L-cysteine (NAC), which is a commonly used ROS scavenger. Treatment with NAC (3 or 6 μM) significantly counteracted the lifespan shortening (Figure 7A; see also Supplemental Figure 3) and lipofuscin accumulation (Figure 7B) mediated by *kin-10* knockdown. To exclude effects of NAC and *kin-10* RNAi treatment on lifespan and lipofuscin accumulation during development, worms were treated with *kin-10* RNAi and NAC only during adulthood. NAC treatment during adulthood only also showed similar counteracting effects on the lifespan shortening and lipofuscin accumulation (Supplemental Figure 4). These findings indicate that in worms, ROS production is one of the major upstream causes of aging induced by CK2 downregulation.

## DISCUSSION

Previous studies demonstrated that the activity of CK2 decreases during replicative senescence in human fibroblast cells and with advancing age in rat tissues [10, 24]. In addition, CK2 inhibition induces premature senescence through the ROS-p53-p21^Cip1/WAF1^ pathway in human cells such as lung fibroblast IMR-90, colon cancer HCT116, and breast cancer MCF-7cells [11–15]. In this study, we examined whether CK2 modulated longevity in *C. elegans*. The present results indicate that CK2 activity decreased with advancing age in *C. elegans* (Figure 1A). Moreover, we showed here that downregulation of CK2 activity by knockdown of *kin-10* (the ortholog of CK2β) shortened the lifespan of *C. elegans* (Figure 1C; see also Supplemental Figures 1 and 2A), induced several age-related biomarkers, increased lipofuscin accumulation (Figure 2A; see also Supplemental Figure 2B) and fat content (Figure 2B), reduced the pharyngeal pumping rate (Figure 1D) and the velocity of body movement (Figure 3C and 3D), and reduced resistance to heat and oxidative stress (Figure 3A and 3B). These events may be consistent with previous findings showing that the capacity for exercise is fundamental to longevity and more notably healthy aging [25], as well as that the accumulation of fat in aged worms may be due to an age-associated decline in tissue function [26]. In addition, CK2 downregulation in *C. elegans* promoted tri-methylation of lysine 9 in histone H3 (H3K9me3), which is a hallmark of senescence associated heterochromatin foci (SAHF) (J.W. Park and Y.S. Bae, manuscript in preparation). It has been reported that SAHF formation represses expression of cell cycle progression associated genes, such as cyclin A2 and transcription factor E2F target genes [27]. How does CK2 activity decrease with advancing age in *C. elegans*? Because our previous studies demonstrated that DNA methylation and microRNAs (miRNAs) are primarily involved in CK2α gene silencing during replicative senescence of human cells [28–30], we speculate that the underlying cause of age-dependent CK2 downregulation in worms may be related to DNA methylation or miRNAs.

Components of IIS, such as PI3K and AKT, have been shown to modulate longevity in yeast, worms, and flies [1–4]. The present study indicated that the lifespan of *age-1* or *akt-1* single mutants was significantly suppressed by *kin-10* RNAi, suggesting that CK2 functioned downstream of AKT in *C. elegans* (Figure 4A and 4B). DAF-16/FoxO is a well-known transducer of IIS [5–7]. The present data showing that the reduced longevity conferred by knockdown of *kin-10* was not additive to the shortened lifespan caused by the *daf-16* mutation suggested that both the *daf-16* mutation and CK2 deficiency acted in the same pathway in *C. elegans* (Figure 4C). We also demonstrated here that CK2 downregulation either decreased or increased the transcriptional activity of DAF-16 depending on the target genes, suggesting that CK2 acts upstream of DAF-16 in *C. elegans* (Figure 5). Thus, we conclude that CK2 functions between AKT-1 and DAF-16 in worms. This is not consistent with our previous report that CK2 acts upstream of the AKT in human colon cancer cells [14, 15]. Although we currently cannot explain this discrepancy, we hypothesize that CK2 may function at different stages of the IIS pathway depending on the organism type (e.g., invertebrate or vertebrate).

A number of studies have shown that increased oxidative damage in intracellular molecules is an important mechanistic explanation for the aging process [31, 32]. A recent paper reported that the effect of antioxidants reveals an inverted U-shaped dose-response relationship between ROS amounts and lifespan in *C. elegans* [33], suggesting that sustaining ROS homeostasis is crucial for maintaining a healthy lifespan. Therefore, intracellular ROS levels should be maintained for preventing aging within narrow ranges by both ROS generating and scavenging systems [32]. We propose here that redox balance can be disrupted by decreased activity of CK2 in *C. elegans*, because *kin-10* knockdown increased ROS levels in worms (Figure 6). Antioxidant NAC successfully counteracted the lifespan shortening and lipofuscin accumulation induced by *kin-10* RNAi, indicating that in worms, ROS generation is a major upstream cause of aging induced by CK2 downregulation (Figure 7; see also Supplemental Figures 3 and 4). The transcriptional activity of DAF-16 on the promoters of *sod-3* and *dod-11* was decreased by *kin-10* RNAi (Figure 5). Since SOD-3/MnSOD detoxifies ROS [34] and DOD-11/sorbitol dehydrogenase reduces the nicotinamide adenine dinucleotide (NAD^+^) level in the conversion of sorbitol to fructose [35], our observations suggest that CK2 inhibition-mediated downregulation of SOD-3 and DOD-11 may cause an imbalance between ROS production and removal in *C. elegans*. These results are consistent with our previous report indicating that CK2 inhibition stimulates ROS production through inhibition of FoxO3a activity in human cells [15]. The present study found that *kin-10* knockdown decreased the transcriptional activity of DAF-16 on the *mtl-1* promoters, but it enhanced that of DAF-16 on the *dod-8* promoter in worms. It has been reported that MTL-1/metallothionein is a heavy metal-binding protein that is involved in metal homeostasis [36] and that DOD-8/17 β-hydroxysteroid dehydrogenase is localized to lipid droplets, which are highly specialized for lipid storage [37]. Thus, this study suggests that CK2 downregulation may reduce stress resistance and increase fat accumulation through controlling DAF-16 activity in worms.

In summary, we uncovered a novel mechanism for regulation of lifespan in *C. elegans*. CK2 downregulation led to shortened longevity, reduced physical ability and stress resistance, and increased lipofuscin accumulation. Importantly, CK2 activity decreased in aged worm. CK2 functioned between AKT and DAF-16 and regulated expression of DAF-16 target genes in worms. Because DAF-16 plays an essential role in the aging process, elucidating the molecular mechanism by which CK2 regulates the transcription activity of DAF-16 will extend understanding of aging processes.

## MATERIALS AND METHODS

### *C. elegans* strains and culture

*C. elegans* N2 (wild-type) strain, strains carrying mutant alleles *daf-16(mu86)*, *age-1(hx546)*, and *akt-1(mg144),* and reporter strains *sod-3::gfp*, *dod-11::gfp*, *dod-8::gfp*, *mtl-1::gfp*, and *daf-16::gfp* (CF1724, *daf-2* mutant) were obtained from the Caenorhabditis Genetics Center. Worms were grown at 20 or 21 °C on nematode growth medium (NGM) agar plates with *Escherichia coli* strain OP50 as a food source. For some experiments, NAC was prepared in dH_2_O and added into NGM media to a final concentration of 3 or 6 µM.

### RNAi experiments

*E. coli* HT115 expressing double-stranded *kin-10* RNA was from the *C. elegans* ORFeome RNAi library. To inactivate *kin-10* function, eggs from gravid adults were placed on HT115-seeded NGM plates and allowed to hatch at 21 °C. Expression of double-stranded RNA was induced by treatment with 1 mM isopropyl 1-thio-β-D-galactopyranoside. Worms hatched from eggs were fed on HT115-seeded NGM plates until L4 stage. To synchronize the worms, L4 larvae were then placed on HT115-seeded NGM plates supplemented with 5 µM 2′fluoro-5′deoxyuridine (FUdR; Sigma, MO), which prevents progeny production, and allowed to grow to day 1 or 8 of adulthood. *E. coli* HT115 carrying the empty L4440 vector was used as control RNAi. For some experiments (Supplemental Figures 2 and 4), we used worms fed with *kin-10* RNAi at the L4 larval stage for 1 day to exclude the effect of *kin-10* knockdown on worm development.

### Lifespan measurement

Lifespan assays were performed as described previously [38]. Synchronized L4 larvae were placed on HT115-seeded NGM plates containing FUdR. Surviving worms were counted daily and were moved to new HT115-seeded NGM plates. Death was scored as the absence of a response to slight touch using a thin platinum wire. Three independent experiments were performed, with approximately 60 worms for each experimental group.

### CK2 activity assay

Worms were lysed by sonication in lysis buffer [50 mM Tris-HCl (pH 8.0), 20 mM NaCl, 1 mM MgCl_2_, 1 mM EDTA, 1% Nonidet P-40, 0.5 mM PMSF, 1 µg/mL aprotinin, 1 µg/mL leupeptin, 1 µg/mL pepstatin, 1 mM sodium orthovanadate, 1 mM sodium pyrophosphate, and 4 mM p-nitrophenyl phosphate]. The particulate debris was removed by centrifugation at 12,000 × *g*. The volumes of the supernatants were adjusted for equal protein concentration. A standard assay for CK2 phosphotransferase activity was conducted in a reaction mixture containing 20 mM Tris-HCl (pH 7.5), 120 mM KCl, 10 mM MgCl_2_, and 100 µM [γ-^32^P]ATP in the presence of 1 mM synthetic peptide substrate (RRREEETEEE) in a total volume of 30 µl at 30 °C. Worm lysates were added to initiate the reactions and incubated for 15 min. The reaction was stopped by the addition of trichloroacetic acid to a final concentration of 10%. The mixture was then centrifuged, after which, 10 µl of supernatant was applied to P-81 paper. The paper was washed in 100 mM phosphoric acid, and radioactivity was measured by scintillation counting.

### Measurement of pharyngeal pumping rate

Synchronized (day 1 and 8 of adulthood) worms were transferred to OP50-lawn on NGM plates, and the pharyngeal pumping rates of individual worms were recorded by counting the number of pharyngeal contractions for 1 min under a dissection microscope.

### Lipofuscin assays

Intestinal lipofuscin was assayed in synchronized (day 1 and 8 of adulthood) worms. The autofluorescence of lipofuscin was measured using a fluorescence microscope (ZEISS AxioCam MRc, Germany) with an excitation/emission wavelength of 350 nm/470 nm. The relative fluorescence intensity was quantified using the ImageJ software (National Institutes of Health, MD) to determine the lipofuscin levels.

### Nile red staining

Nile red staining was performed as previously described [39], with minor modifications. Briefly, synchronized (day 1 and 8 of adulthood) worms were grown on HT115-seeded NGM plates containing 75 ng/mL of Nile red (Sigma, MO) for 3 days. Worms were mounted on a 2% agarose pad and visualized with a fluorescence microscope (Carl Zeiss AxioScope A1) at 10× magnification. The data were quantified using the ImageJ software by determining the average pixel fluorescence intensity. The relative fluorescence of the whole body was determined densitometrically using the Image-Pro Plus version 6.0 software (Media Cybernetics, MD).

### Measurement of mean velocity and maximum velocity

The worms’ mean velocity and maximum velocity were measured as previously described [40]. Briefly, synchronized (day 1 and 8 of adulthood) worms were transferred to a physical assay plate (NGM plate without bacterial lawn), and their movements were recorded immediately. The recording system comprised a stereomicroscope (Nikon SMZ 745T), a charge-coupled device camera (TUCSEN TCH-5.0), and imaging software (TUCSEN ISCapture). The recording period was 30 s at a rate of 30 frames per second. The locomotion velocity was expressed as millimeter per second [the distance (mm) between displaced centroids per second]. Recorded images were analyzed by ImageJ and wrMTrck (plugin for ImageJ: www.phage.dk/plugins). The locomotion velocity data were imported into an Excel spreadsheet. The peak locomotion velocity observed in the 30 s period was used as the maximum velocity.

### Stress resistance assays

Thermotolerance assays were performed as previously described [41]. Briefly, HT115-seeded NGM plates with synchronized (day 1 and 8 of adulthood) worms were shifted from 21 °C to 35 °C. After the temperature shift, animal survival was scored for touch-provoked movement every 2 h. For the oxidative stress resistance assay, synchronized (day 8 of adulthood) worms were transferred to HT115-seeded NGM plates containing 3 mM of H_2_O_2_. After 5 h of incubation, worm viability was scored as touch-provoked movement every 4 h. Data were analyzed and plotted as described for the lifespan assays.

### Measurement of intracellular ROS

Synchronized (day 1 and 8 of adulthood) worms were washed with M9 buffer (22 mmol/L KH_2_PO_4_, 22 mmol/L Na_2_HPO_4_, 85 mmol/L NaCl, and 1 mmol/L MgSO_4_) and then transferred to 2 mL of M9 buffer containing 6 µM dichlorofluorescein diacetate (DCFDA, Invitrogen, Carlsbad, CA) or 3 µM dihydroethidium (DHE, Invitrogen, Carlsbad, CA). After incubation for 60 min at 37 °C in the dark, the worms were rinsed with M9 buffer, and digital images were obtained with a Leica DM IRB inverted microscope (Leica Microsystems, Germany) equipped with a CoolSNAP HQ camera (Roper Scientific, NJ) operated by the Metamorph Image Software (Universal Imaging Corporation, PA).

### Reporter gene assay

Synchronized (L4 larva) reporter worms *dod-8::gfp*, *dod-11::rfp*, *mtl-1::rfp,* and *sod-3::gfp* were transferred to HT115-seeded NGM plates. After 1 day, the fluorescence of GFP or RFP was measured using a fluorescence microscope (ZEISS AxioCam MRc, Germany) at an excitation/emission wavelength of 490 nm/525 nm. The relative fluorescence intensity was quantified using the ImageJ software to determine GFP levels.

### Statistical analysis

The statistical significance of differences in the data was analyzed by one-way ANOVA with the SPSS package program. The results were considered significant if the value of *P* was less than 0.05. The Duncan's multiple-range test was performed if differences between groups were identified as α = 0.05.

## SUPPLEMENTARY MATERIALS FIGURES


