# Supplementary material for: Downregulation of protein kinase CK2 activity induces age-related biomarkers in C. elegans
Source: Oncotarget. 2017 Apr 7;8(23):36950–63. doi: 10.18632/oncotarget.16939 (PMC5513713; doi:10.18632/oncotarget.16939)
Supplement: Supplementary file 1 [file oncotarget-08-36950-s001.pdf]

Downregulation of protein kinase CK2 activity induces age-related biomarkers in *C. elegans*

Supplementary Material

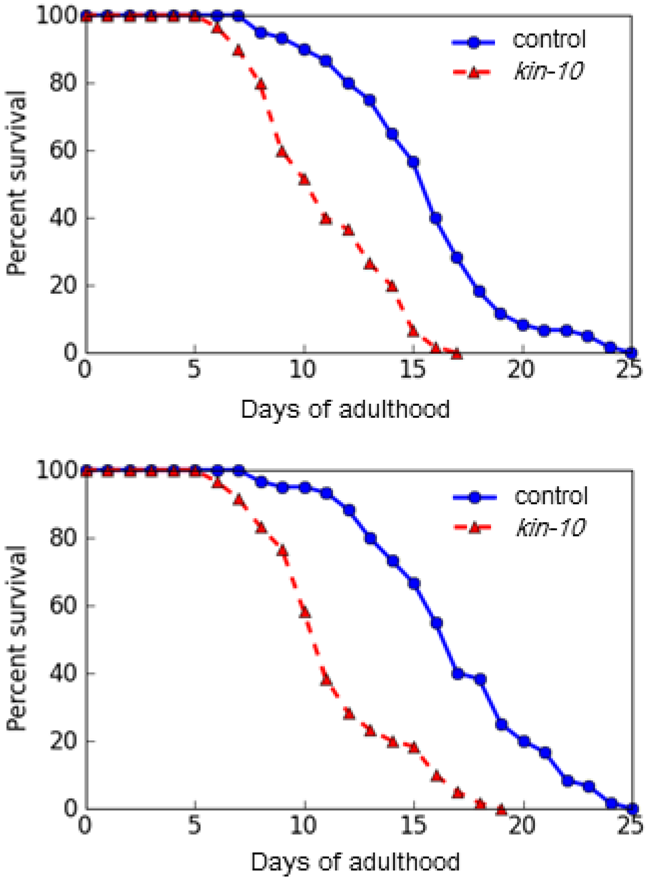

Supplemental Figure 1: Effect of kin-10 RNAi on the lifespan of worms. Age-synchronized L4 larvae were fed on empty vector control (L4440) or *kin-10* RNAi plates under standard conditions. Viability was scored as movement away from pick touch at the indicated days. Representative data from three independent RNAi experiments are shown (n = 60 per condition). All experiments in this figure were carried out at 21 °C.

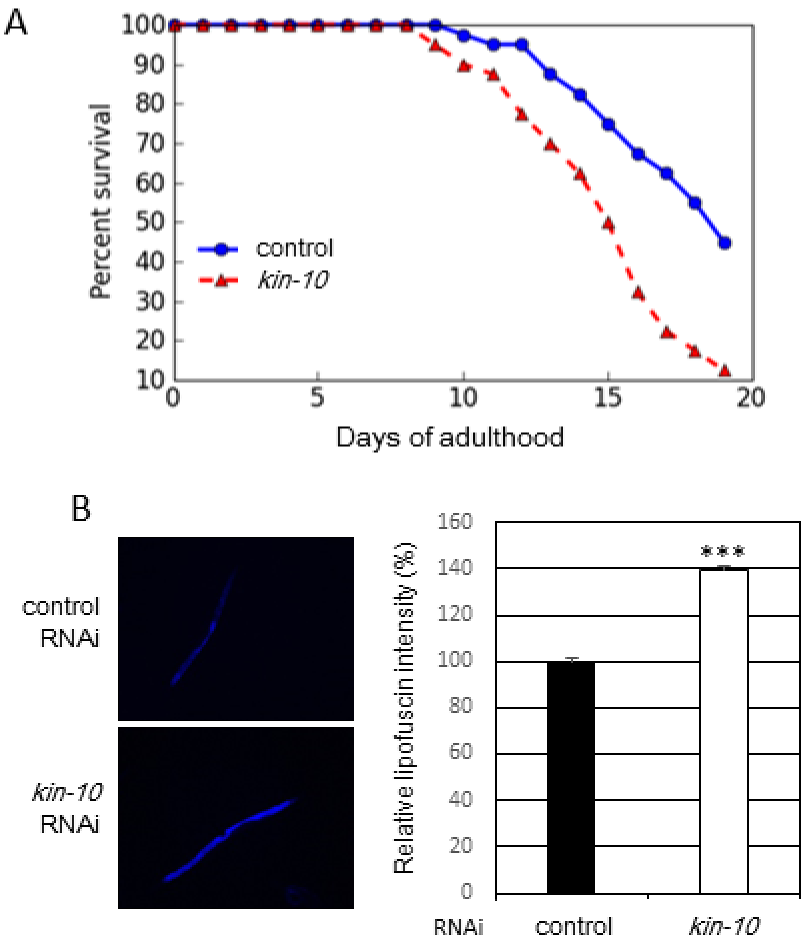

Supplemental Figure 2: Knockdown of kin-10 using RNAi only during adulthood also reduced lifespan and increased lipofuscin accumulation. Worms were fed on empty vector control (L4440) or *kin-10* RNAi plates only at the L4 larval stage for 1 day. A. *Kin-10* knockdown during adulthood decreased lifespan. Viability of worms was scored as movement away from pick touch at the indicated days. Representative data from three independent RNAi experiments are shown (n = 40 per condition). B. *Kin-10* knockdown during adulthood stimulated lipofuscin accumulation. Representative autofluorescence images of worms are shown (left panel). The fluorescence intensity (right panel) was quantified using the ImageJ software by determining the average pixel intensity (n > 60 per condition). Values indicate mean ± SEM. ****P* < 0.001. All experiments in this figure were carried out at 20 °C.

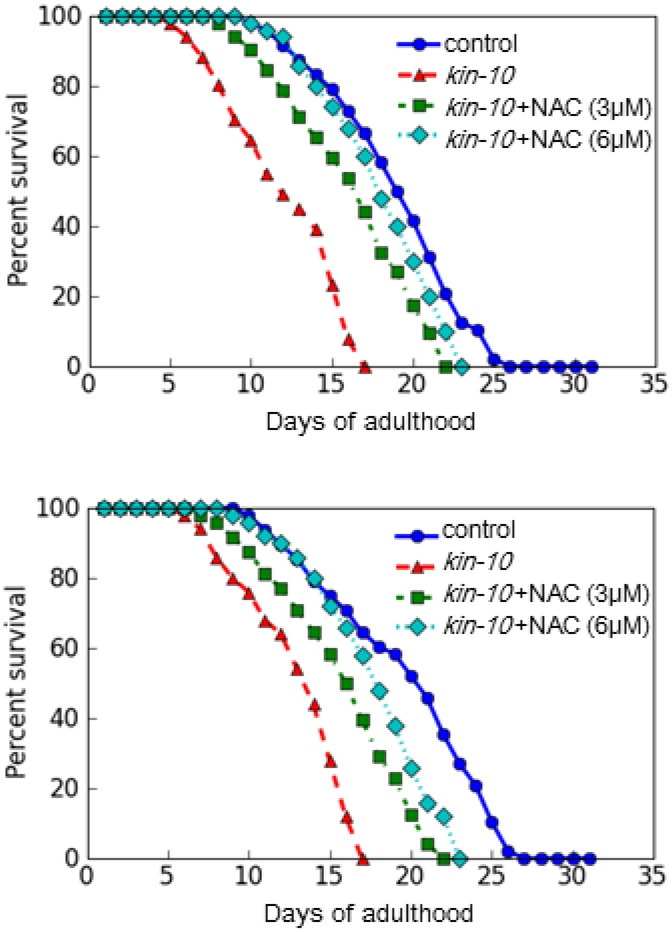

Supplemental Figure 3: Effect of NAC on lifespan shortening induced by kin-10 RNAi. Age-synchronized L4 larvae were fed on empty vector control (L4440) or *kin-10* RNAi plates containing 0, 3, or 6 µM NAC (n = 50 per condition). Viability was scored as movement away from pick touch at the indicated days. All experiments in this figure were carried out at 21 °C.

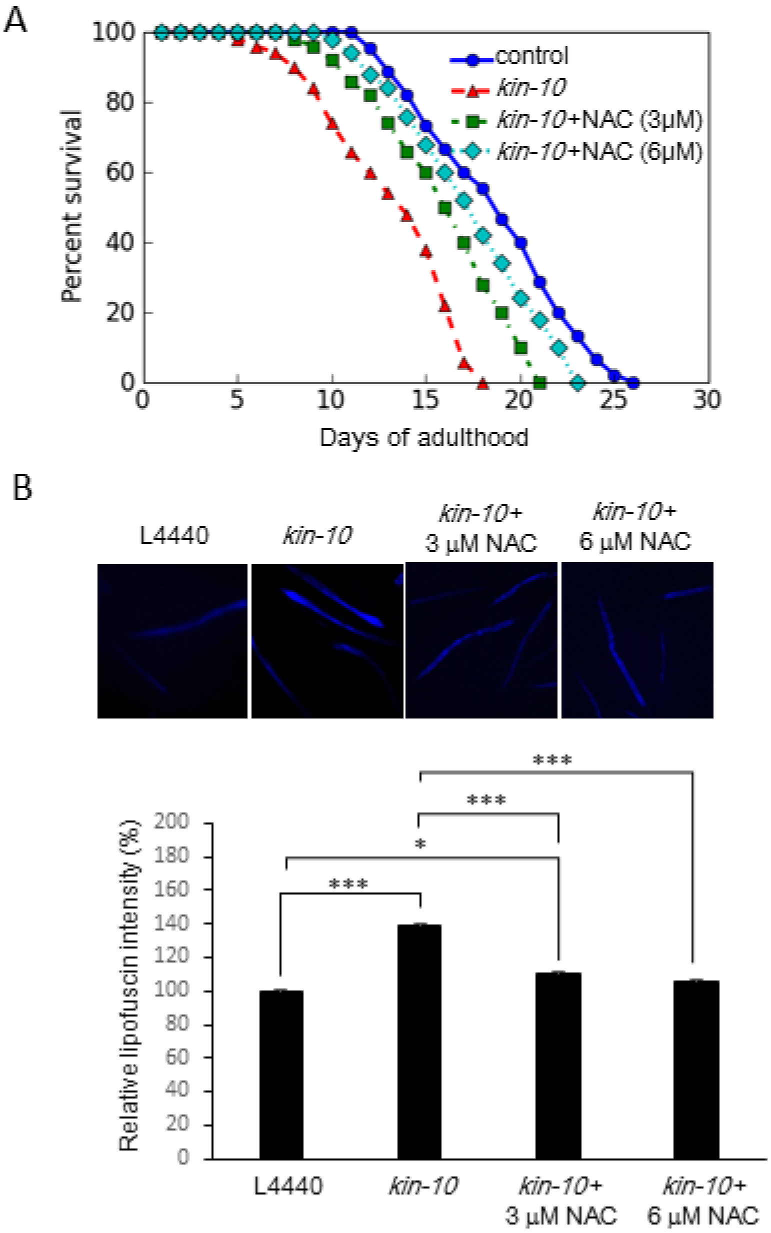

Supplemental Figure 4: Knockdown of kin-10 using RNAi only during adulthood also reduced lifespan and increased lipofuscin accumulation. Worms were fed on empty vector control (L4440) plates or *kin-10* RNAi plates containing NAC (0, 3, or 6 μM) only at the L4 larval stage for 1 day. A. Viability of worms was scored as movement away from pick touch at the indicated days. Representative data from three independent RNAi experiments are shown (n = 50 per condition). B. Representative autofluorescence images of worms are shown (upper panel). The fluorescence intensity (bottom panel) was quantified using the ImageJ software by determining the average pixel intensity (n > 36 per condition). Data are shown as the means ± SEM. *$P < 0.05$; ***$P < 0.001$. All experiments in this figure were carried out at 21 °C.
